# Improving the Metabolic and Mental Health of Children with Obesity: A School-Based Nutrition Education and Physical Activity Intervention in Wuhan, China

**DOI:** 10.3390/nu12010194

**Published:** 2020-01-10

**Authors:** Hong-jie Yu, Fang Li, Yong-feng Hu, Chang-feng Li, Shuai Yuan, Yong Song, Miaobing Zheng, Jie Gong, Qi-qiang He

**Affiliations:** 1 School of Health Sciences, Wuhan University, Wuhan 430071, China; 2 Wuhan Center for Disease Prevention and Control, Wuhan 430022, China; 3 Xinzhou Center for Disease Prevention and Control, Wuhan 431400, China; 4 Unit of Cardiovascular and Nutritional Epidemiology, Institute of Environmental Medicine, Karolinska Institutet, Nobelsväg 13, 17177 Stockholm, Sweden; 5 Institute for Physical Activity and Nutrition, School of Exercise and Nutrition Sciences, Deakin University, Geelong, VIC 3125, Australia; 6 Hubei Biomass-Resource Chemistry and Environmental Biotechnology Key Laboratory, Wuhan University, Wuhan 430071, China

**Keywords:** childhood obesity, nutrition education, physical activity intervention, metabolic health, mental health

## Abstract

This study aimed to evaluate the effectiveness of a school-based nutrition education and physical activity intervention on cardiovascular risk profile and mental health outcomes among Chinese children with obesity. Two primary schools were randomly allocated to the control group (CG) and the intervention group (IG). We selected children with obesity from 1340 students in the third and fourth grades as participants. The IG received 8 months of nutrition education and physical activity intervention, while the CG was waitlisted. A generalized estimating equation model was applied to assess repeated variables over time. A total of 171 children with obesity (99 IG and 72 CG) aged 9.8 ± 0.7 years completed the post-intervention stage. Compared with baseline, significant reductions were observed within the IG for depression and fasting plasma glucose at post-intervention. After adjusting for confounders, group and time interaction effects showed that the IG achieved improvements in the risk of poor well-being (*p* = 0.051) and social anxiety (*p* = 0.029), had decreased diastolic blood pressure (*p* = 0.020) and fasting plasma glucose (*p* < 0.001), and had significantly increased high-density lipoprotein (*p* < 0.001) from baseline to post-intervention relative to the CG. The effects of school-based nutrition education and physical activity intervention on children with obesity are diverse, including not only the improvement of metabolic health but also mental health promotion.

## 1. Introduction

Childhood obesity is a serious public health challenge worldwide. The global prevalence of overweight and obesity combined for children rose by 47.1% between 1980 and 2013 [1]. China is one of the largest contributors, accounting for 5.1% (15.4 million individuals) of global obesity in 2015 [2]. Meanwhile, the National Surveys on Chinese Students’ Constitution and Health reported that 20.3% of children aged 7–12 years suffered from obesity [3].

As a result of possessing unhealthy lifestyle factors such as poor diet and lack of physical activity, children with obesity have increased risks for metabolic diseases and poor mental health [4,5]. Recent systematic reviews indicated that the proportion of individuals with three or more abnormal metabolic indicators from blood test was much higher among children with obesity than normal-weight children (61.1% vs. 0.6%) [4]. Moreover, children with obesity were more likely to suffer from depression (Odds ratio, OR = 1.46) and anxiety (OR = 1.47) [6]. The prevalence of depression and anxiety among children/adolescents with overweight or obesity in China is alarming, at 21.7% and 39.8%, respectively [6]. Besides, childhood obesity has been linked with several chronic diseases in adulthood [7]; hence, early treatment interventions are urgent for promoting the metabolic and mental health of children with obesity.

Despite numerous studies having explored the effectiveness of school-based childhood obesity interventions, results on the cardiovascular risk profile have been inconsistent [8,9]. A meta-analysis showed differential intervention effects on anthropometric and blood lipids outcomes, with more than half of 17 studies showing no significant effect on either, while three of interventions demonstrated beneficial changes on both [9]. In addition, most of the previous studies were lifestyle interventions to prevent the incidence of obesity in the general population rather than to study the treatment effect on children with obesity [10,11]. A meta-analysis of 76 school-based obesity intervention studies in mainland China revealed that more than 80% of the studies were of poor quality and were published in Chinese. Moreover, limited studies utilized both nutrition education and physical activity as intervention strategies [12]. The effectiveness of school-based obesity interventions with focuses on both nutrition education and physical activity targeting children with obesity on obesity outcomes and cardiovascular risk profile among Chinese population is therefore unclear. Furthermore, the school-based intervention studies on mental health among children with obesity are scarce. Given the high prevalence of mental health problems for children with obesity, interventions to improve mental health tailored specifically for this population are warranted [6]. Emerging studies suggest that the mental health of children with obesity was significantly linked with lifestyle factors such as dietary intake and physical activity [13,14,15]. We therefore hypothesize that school-based interventions involving nutrition education and physical activity may also be effective in promoting mental health among Chinese children with obesity.

Considering the double burden of obesity prevalence in metabolic and mental health problems among Chinese school-aged children with obesity, it is imperative to come up with appropriate school-based intervention strategies and test their effectiveness in improving obesity prevalence and metabolic and mental health outcomes. We therefore conducted a nutrition education and physical activity intervention among Chinese school-aged children with obesity and examined its effect on obesity outcomes (body mass index, BMI; waist circumference, WC), cardiovascular risk profile (blood pressure, BP; fasting blood glucose, FPG, and lipids) and mental health outcomes (poor well-being, depressive symptoms and social anxiety).

## 2. Materials and Methods

### 2.1. Study Design

A school-based nutrition education and physical activity was conducted from November 2015 to June 2016 in Wuhan, China. The study was approved by the Medical Research Ethics Committee of Wuhan University and was registered with ClinicalTrials.gov (ID: NCT02773823). Written informed consent was obtained from parents of the children. Two primary schools with a similar scale of size were selected from Xinzhou District, Wuhan. The schools were randomly allocated to the intervention group (IG) or control group (CG).

### 2.2. Study Subjects

**Figure 1:**
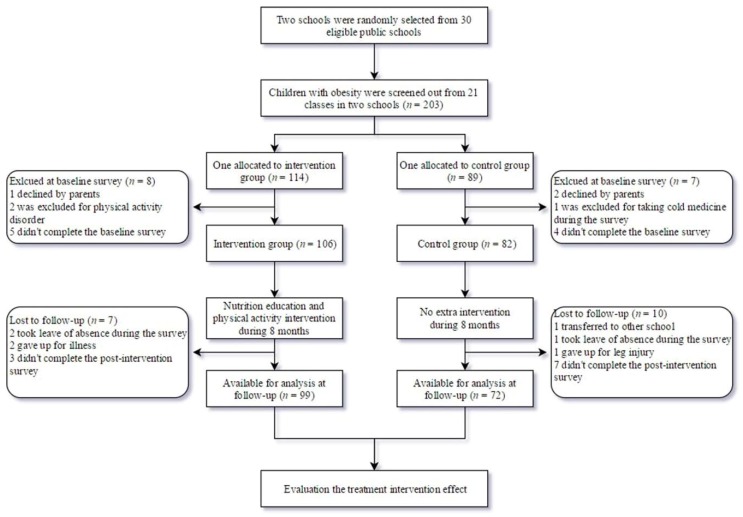
Participants flow diagram.

All students in the third and fourth grades were screened for obesity by measuring their height and weight. BMI, calculated by weight (kg) divided by height (meters) squared, was used to identify children with obesity based on Chinese childhood obesity BMI (kg/m^2^) cut-off points (8-year-olds: ≥ 20.3 for boys and ≥ 19.9 for girls; 9-year-olds: ≥ 21.4 for boys and ≥ 21.0 for girls; 10-year-olds: ≥22.5 for boys and ≥ 22.1 for girls; 11-year-olds: ≥ 23.6 for boys and ≥ 23.3 for girls) [16]. Children who suffered from serious illness (such as heart diseases, diabetes, and asthma, etc.) and mental disorders, or could not participate in physical exercise were excluded from this study. Sample size was calculated by the equation: *N* = 2σ^2^(Z_α_ + Z_β_)/Δ^2^, where *N* = estimated sample size, σ = standard deviation for BMI = 0.10, and ∆ = tolerable difference = 0.03, with the Z statistic corresponding to a chosen level of confidence (Z_α_ = 1.96 for α = 0.05; Z_β_ = 0.84 for β = 0.20) [17]. The sample size required for this study was 63 for each group. Considering a follow-up lost rate of 10%, the required sample size increased to 70. Among 203 eligible children with obesity, 188 participated in the baseline study and 171 (99 IG and 72 CG) completed the whole study. Seventeen children were excluded mainly due to school transfer, leave of absence, illness or injury during the survey, failure to complete the post-intervention survey for having breakfast before the blood test, missing rate of the questionnaire items over than 10%, or major life changes (parents’ divorce or death of relatives). Figure 1 presents the participants flowchart.

### 2.3. Intervention

Following a previous obesity intervention study in Chinese school children/adolescents [18], the design of present intervention content followed the same process: (1) Social and epidemiological assessment, (2) Educational and ecological assessment, (3) Administrative and policy assessment, (4) Pilot study. Due to the small sample size, we did not conduct a pilot study. Three main phases of intervention implementation and strategies are listed in Table 1.

Firstly, we created a supportive school and family environment. It has been well documented that family and school environments are ecological models for the development of healthy lifestyle during childhood [19]; thus the agent of intervention delivery included caregivers and teachers. Teachers cooperated with parents to supervise the implementations of intervention content at school and home. Parents were invited to participate in health education curriculums and exercise at home with their children together.

Secondly, we implemented compulsory daily exercise on school day and lifestyle modification on participants. It consisted of a 20-min class recess in the morning in the form of jogging. One extra gym class (40 min) after school in the afternoon included three forms of exercises (rope skipping, badminton, and 200-m relay race). The modification of lifestyle was mainly performed through nutrition and physical activity education classes. The themes of nutrition education were to promote the intake of healthy food and developing healthy dietary habits. Meanwhile, physical activity education was provided to children in the IG to aim at increasing physical activity and decreasing sedentary behavior. The classes were scheduled as four sessions (once every two months) of 60 min each. In addition, setting multiple goals (at least five portions of fruits and vegetables daily consumption, no more than 2 h/day on screen viewing, etc.) and leading peer competitions were adopted to strengthen the cultivation of healthy lifestyles [20].

Finally, a series of strategies were applied to control the intervention quality, including the training for project members, immediate revising intervention measures based on the feedback from children, parents and teachers, and diversifying the forms of intervention to attract children and improve their subjective initiative.

Parents of children in the CG received the results of anthropometric and blood test results and simple suggestions about healthy lifestyles to reduce their child’s body weight. Apart from that, Children in the control school followed their usual practice with no extra intervention.

### 2.4. Measures

At the start and the end of the intervention study, anthropometric parameters, blood pressure measurements, and fasting blood tests were conducted by a team of trained investigators in the classroom with air-conditioner to keep comfort temperature. Children were asked to report their frequency of several lifestyles and psychological status using a self-administered questionnaire. All items were read by the investigator slowly and explained the definition of some words in the classroom. Two investigators checked the completeness of the questionnaire. Children’s parents provided their household income level.

*Anthropometric parameters and blood pressure measurement*: The children’s height (without shoes) and weight (in light clothes) and WC were measured using standard methods [21]. Blood pressure, including systolic blood pressure (SBP) and diastolic blood pressure (DBP) were measured with all children sitting in an upright position for at least 5 min. Two measurements were taken in the morning, and the mean was used for data analysis.

*Fasting blood test*: Blood samples were taken from the antecubital vein after an overnight fast and were tested in a standardized laboratory of Xinzhou Center for Disease Control and Prevention. FPG, triglyceride (TG) and High-density lipoprotein (HDL) were analyzed enzymatically with a Mairui BS-300 Automatic Analyzer (Mairui High Technologies Corp).

*Psychological factors*: The methods for assessing well-being and depression of children with obesity have been described in the previous research [14]. Social anxiety was identified using the Social Anxiety Scale for Children (SASC), which was developed to evaluate children’s feelings of social anxiety in the context of their peer relations [22]. It consists of 10 items that assess social avoidance and distress and fear of negative evaluation. A total score ≥ 7 indicates social anxiety. The SASC has been examined in Chinese children and showed acceptable reliability and validity [23].

### 2.5. Statistical Analysis

Obesity outcomes (BMI, WC), cardiovascular risk profile (BP, FPG and lipids) and mental health outcomes (poor well-being, depressive symptoms and social anxiety) between the IG and CG were examined by using Mann–Whitney U test (for continuous variables) or χ^2^ test (for categorical variables), where appropriate. However, the baseline and post-intervention were interrelated within the group. Thus, the Wilcoxon signed-rank test (for BMI and metabolic parameters) and McNemar’s test (for behaviors and mental health parameters) were applied to examine changes from baseline to post-intervention within the CG and IG, respectively. To account for the effects of potential confounders, generalized estimating equation (GEE) models with the exchangeable structure were used to assess the repeated variables. Gamma GEE with log link was applied to skewed continuous variables (BMI, SBP, DBP, FPG, TG, and HDL) and binary logistic GEE was applied to classified variables (poor well-being, depression, and social anxiety). Statistical analyses were conducted using the SPSS statistical package (Version 23.0; SPSS Inc. Chicago, IL, USA).

## 3. Results

The basic characteristics of participants are shown in Table 2. Although children in the CG had a significantly higher height than those in the IG, no significant differences in sex ratio, age, weight, and monthly household income level were found between the two groups.

Table 3 displays the comparisons of different health outcomes between CG and IG. Regardless of the similar significant changes of WC and BP between the two groups, a significant reduction of FPG was observed in the IG from baseline to post-intervention, while that in the CG increased significantly. Although the increase of TG in the IG was significant, it was still lower than that of CG in the post-intervention. Similarly, the mean FPG in the IG was significantly lower than that of CG in the post-intervention. There was no significant difference in psychological factors between the two groups in the baseline and post-intervention. However, after the 8-month comprehensive intervention, the proportion of poor well-being (*p* = 0.001) and depression (*p* = 0.064) in the IG decreased from baseline to post-intervention.

Table 4 shows the results of the GEE analyses results for different health outcomes. The IG achieved significant decreases in SBP (*p* < 0.001)) and FPG (*p* < 0.001) and a significant increase in HDL (*p* < 0.001) compared with the CG through the group*time interaction term. In the meantime, the IG demonstrated a marginally significant improvement for poor well-being (*p* = 0.051) and a decreased risk for self-reported social anxiety (*p* = 0.029) using the group * time interaction term.

## 4. Discussion

Findings from this study showed that the school-based nutrition education and physical activity intervention in Chinese children with obesity did not significantly decrease BMI and WC in the IG compared with CG. However, the interventions did effectively reduce the risk of some metabolic abnormalities, poor well-being and social anxiety among participants.

With regard to the slightly increased BMI and WC after the intervention, our study was consistent with another obesity treatment intervention on metabolic profile in China [24]. However, effects on BMI and WC outcomes were still controversial among school-based childhood obesity prevention programs. In a meta-analysis of 27 childhood obesity prevention programs, Huang et al. found a limited efficacy of intervention in improving BMI or skinfold thickness [11]. While several other systematic reviews and meta-analyses still showed positive effects on the change of BMI and WC among children [25,26,27]. It seems logical that BMI and WC still keep uptrend in the progress of the intervention because children are at the peak period of growth and development [24]. Therefore, it may underestimate the effectiveness of intervention studies when simply using anthropometric indicators as independent outcomes. Thus, future intervention studies should explore more accurate indicators to assess the effect of the interventions on childhood obesity.

Our study showed that BP decreased from baseline to post-intervention in both the CG and IG, which aligned with two previous intervention trials conducted by Almas et al. [28] and Seo et al. [29]. A previous study suggested that BP measurement underwent a seasonal variation or influenced by outdoor temperature [30]. The BP of our study was assessed in October and June, respectively. Therefore, measurement bias could not be completely excluded. Additionally, the group*time interaction results indicated the mean change of SBP, rather than DBP, from baseline to post-intervention was significantly different between two groups, which were in agreement with the findings from Almas’ study [28], but inconsistent with Seo’s study [29]. It is likely that these differences may result from the different duration and intensity of intervention [8]. Hence, long-term and rigorously designed interventions are needed to further assess the effect on BP of children with obesity.

The significant improvement of the blood lipid profile has also been reported in previous intervention studies [8,9,31,32]. Two recent systematic review and meta-analysis showed that fewer than half of the included studies reported the improvements of HDL and FPG [9,32]. Likewise, in the present study, the nutrition education and physical activity intervention posed a significant improvement in HDL and a decrease in FPG among schoolchildren with obesity. HDL has the ability to remove “harmful” cholesterol in surrounding tissues, thereby converting them to bile acids or excreting directly from the gut through bile while elevated FPG can easily increase the risk of insulin resistance [31]. Animal study explored the mechanism of combining exercise with diet control on lipid metabolism was through gastrointestinal hormones to optimize the process of digestion and absorption of nutrients by the gut or through AMP-activated protein kinase (AMPK) to maintain the balance of FPG [33]. However, the difference between two groups in the change of TG was insignificant, other studies also did not observed the effects on TG [34,35]. Ho et al. compared the effect of a dietary and exercise intervention on the risk of metabolism and finally found that the diet-only intervention led to a greater reduction in TG [32]. Unlike the included studies, our study only implemented nutrition education on participants, and lacked the effective control of daily eating habits and restriction of total energy intake, which might affect the non-significant effects. In addition, small sample size may also influence the variation in results [35]. Thus, high-intensity interventions with a higher number of participants need to be explored in future obesity intervention studies.

Mental disorder is a common comorbidity of obesity [36,37]. However, the effects of lifestyle interventions on children’s mental health outcomes have been understudied. Our study is one of the few studies that assessed the potential mental benefits of the school-based intervention among children with obesity. In a systematic review of fourteen physical activity and exercise interventions on childhood overweight and obesity, Ruotsalainen found positive effects of the intervention on self-perception, body satisfaction and eating disorder symptoms, but not on depressive symptoms [38]. A review of seven community-based obesity prevention programs by Hoare et al. summarized positive outcomes in mental health, including modestly decreased anxiety symptoms and increased health-related quality of life in IG [39]. Similarly, we found decreased risk (albeit insignificant) of depression, as well as significantly lower risk for poor well-being and social anxiety in the IG. However, we also observed that the risk of poor well-being and depression in the CG decreased slightly. The possible reason might be that the parents of children in CG had received the anthropometric and blood test results and healthy lifestyle suggestions, and then paid more attention to their child’s health. It has been reported that parent’s perception on children’s weight status played a precursor role for preventive action [40]. Although the mechanisms of intervention affecting psychological outcomes are yet to be clarified, we recommend that future studies should include psychological indicators as primary outcomes for measuring the effect of childhood obesity intervention.

The present study has several limitations. First, mental health status was self-reported. Thus, recall bias cannot be ruled out. Nevertheless, standardized questionnaires that measured the children’s mental health have been used previously and showed acceptable results in Chinese schoolchildren. Second, the relatively short duration and low-intensity dietary intervention of the study may have limited the effectiveness of the intervention. Therefore, a longer period of study with high-intensity diet plus exercise intervention is warranted to explore the more positive influence on health outcomes. Last, all participants were recruited from two schools in one district, which resulted in a wide difference in the numbers of males and females, and limited the generalization of our findings to the national level.

## 5. Conclusions

In summary, findings from this study showed that school-based obesity interventions with focus on nutrition education and physical activity promotion can improve poor well-being and social anxiety and lead to favorable changes in metabolic health among Chinese children with obesity. However, further long-term high-quality school-based intervention studies are desirable to confirm these findings. Future intervention studies should integrate psychological practice as part of intervention strategies to improve mental health and combat childhood obesity.

## Figures and Tables

**Table 1:** Details of intervention strategies.

Content	Targets	Concrete Techniques	Periods	Agent of Delivery
**Supportive environment**	Obtain teacher’s cooperation and supervision	(1) Understand the teaching plan for setting an appropriate exercise period to avoid interrupting the teaching plan and children’s academic achievements in the final exam. (2) Ensure the necessity of exercise facilities (rope and badminton, etc.). (3) Give instructions to let them know what, how and when to monitor the intervention on participants.	Initial stage	Designer and core members
	Obtain parental participation and supervision	(1) Give a lecture for parents to learn the prevalence, cause, and harms of childhood obesity. (2) Deliver what needs to be supervised to parents, including limitation of screen time, preparation of a balanced diet, restricting intake of sugar-sweetened beverages, and ensuring adequate sleep and regular measurement of children’s body weight. (3) Encourage them to participate in health education lecture in class and physical activity at home with children.	Initial stage	Project members
	Advance health propaganda	(1) Chinese dietary guidelines including the food guide pyramid. (2) Physical activity guidelines.	Whole intervention	Project members
**Intervention implement**	Compulsory exercise	(1) Jogging 20 min in the morning break every weekday. (2) Rope skipping 40 min on Monday and Thursday; play badminton 40 min on Wednesday and Friday; 200-m relay race 40 min on Tuesday.	Except the last month before final exam	Gym teachers
	Lifestyle modification	(1) Health education class for children and parents in the class.Benefits of healthy lifestyles (physical activity and balanced diet); Harm of unhealthy lifestyles (sedentary behavior, western food, eating too fast, and skipping breakfast); How to persist in healthier habits and resist the temptation of unhealthy lifestyles.	Once every two months	Core members
		(2) Self-reported unhealthy lifestyles and setting goals to change them, sharing the goal with teachers and parents to supervise.		Teachers and parents
**Quality control**	Training	(1) Before the intervention, ensure all the project members experienced specialized training, including lecture skills, questionnaire survey method, and measurement of anthropometrics. (2) All the cardiovascular risk profiles were measured by professionals from the Centers for Disease Prevention and Control.	Initial stage	Designer and core members
	Feedback	(1) Revise the intervention strategy according to the feedback from teachers and parents, especially the obstacles and success factors.	Once every two months	Core members
	Improve the education method	(1) Health education as much as possible in the form of animation to make it more attractive to children. (2) Use some games to review the health education context. (3) Adopt incentives to individuals who achieve the self-reported goals.	Whole intervention	Core members

**Table 2:** Basic characteristics of participants at baseline.

Characteristics	Total (*n* = 171)	CG (*n* = 72)	IG (*n* = 99)	*p* Value ^a^
Boys, *n* (%)	136(79.5)	54(75.0)	82(82.8)	0.251
Age, years	9.8(0.7)	9.7(0.6)	9.9(0.7)	0.084
Height, cm	144.8(5.8)	145.8(5.7)	144.0(5.8)	**0.041**
Weight, kg	51.2(7.2)	51.9(6.7)	50.6(7.5)	0.119
Monthly household income ^b^, *n* (%)				
Low (<5000 Yuan RMB)	71(43.8)	27(42.2)	44(44.9)	0.383
Middle (5000–10,000 Yuan RMB)	67(41.4)	30(46.9)	37(37.8)	
High (>10,000 Yuan RMB)	24(14.8)	7(10.9)	17(17.3)	

Note: Boldface indicates statistical significance (*p* value < 0.05). Values are M (SD) for continuous variables or *n* (%) for classified variables. CG, Control group; IG, Intervention group. ^a^ Mann–Whitney U (continuous variables) and Chi squared test (classified variables) between the CG and IG. ^b^ Missing value: Monthly household income (*n* = 9).

**Table 3:** Comparisons of different health outcomes between the control and intervention group.

Outcomes	CG (*n* = 72)		*p* Value ^a^	IG (*n* = 99)		*p* Value ^b^	*p* Value ^c^	*p* Value ^d^
	Baseline	Post		Baseline	Post			
**Anthropometric indicators, mean (SD)**								
BMI, kg/m^2^	24.3(1.9)	24.5(2.4)	0.346	24.3(2.5)	24.4(2.7)	0.348	0.585	0.675
WC, cm	82.4(5.7)	85.1(5.8)	**<0.001**	81.5(6.6)	84.0(10.1)	**<0.001**	0.651	0.468
**Cardiovascular risk profile, mean (SD)**								
SBP, mmHg	107.0(10.9)	102.1(8.9)	**0.006**	107.3(10.3)	102.7(8.1)	**<0.001**	0.095	0.606
DBP, mmHg	71.3(7.0)	68.5(6.5)	**0.002**	74.6(8.0)	67.4(6.1)	**<0.001**	**0.017**	0.275
FPG, mmol/L	4.6(0.5)	5.1(0.4)	**0.002**	5.0(0.4)	4.6(0.5)	**<0.001**	<0.001	**<0.001**
TG, mmol/L	1.2(0.6)	1.3(0.6)	0.142	1.1(0.6)	1.2(0.6)	0.023	0.694	0.216
HDL, mmol/L	1.4(0.2)	1.3(0.2)	0.955	1.4(0.3)	1.5(0.3)	0.228	0.672	**0.015**
**Mental health, *n* (%)**								
Poor well-being	18(25.0)	13(18.0)	0.359	27(27.3)	13(13.1)	**0.001**	0.739	0.376
Depression	16(22.2)	13(18.0)	0.648	27(27.3)	16(16.2)	0.064	0.452	0.881
Social anxiety	16(22.2)	20(27.8)	0.388	30(30.3)	20(20.2)	0.137	0.239	0.321

Note: Boldface indicates statistical significance (*p* value < 0.05). Values are mean (SD) for continuous variables or *n* (%) for classified variables. CG, Control group; IG, Intervention group; BMI, Body mass index; WC, Waist circumference; SBP, Systolic blood pressure; DBP, Diastolic blood pressure; FPG, Fasting plasma glucose; TG, Triglyceride; HDL, High-density lipoprotein; SD, Standard deviation. ^a^ Wilcoxon signed-rank (continuous variables) and McNemar’s test (classified variables) between baseline and post-intervention for the CG. ^b^ Wilcoxon signed-rank (continuous variables) and McNemar’s test for (classified variables) between baseline and post-intervention for the IG. ^c^ Mann–Whitney U (continuous variables) and Chi squared test (classified variables) between the CG and IG at baseline. ^d^ Mann–Whitney U (continuous variables) and Chi squared test (classified variables) between the CG and IG at post-intervention.

**Table 4:** Regression analysis of different outcomes before and after intervention.

Outcomes	Time, Post			Group, Intervention			Group * Time		
	β × 10	95% CI	*p* ^c^	β × 10	95% CI	*p* ^c^	β × 10	95% CI	*p* ^c^
**Anthropometric indicators ^a^**									
**BMI, kg/m^2^**	0.08	(−0.03, 0.18)	0.152	0.05	(−0.24, 0.35)	0.720	0.01	(−0.12, 0.14)	0.886
WC, cm	0.30	(0.17, 0.42)	**<0.001**	0.01	(−0.23, 0.24)	0.946	−0.10	(−0.39, 0.18)	0.478
**Cardiovascular risk profile ^a^**									
SBP, mmHg	−0.41	(−0.70, −0.12)	**0.005**	0.47	(0.17, 0.78)	**<0.001**	−0.66	(−1.03, −0.29)	**<0.001**
DBP, mmHg	−0.42	(−0.74, −0.11)	**0.008**	0.12	(−0.16, 0.41)	0.401	0.01	(−0.37, 0.38)	0.972
FPG, mmol/L	0.02	(0.00, 0.04)	**0.039**	0.06	(0.03, 0.09)	**<0.001**	−1.24	(−1.52, 1.43)	**<0.001**
TG, mmol/L	1.34	(0.05, 2.62)	**0.042**	−0.07	(−1.53, 1.38)	0.922	−0.02	(−1.45, 1.40)	0.975
HDL, mmol/L	−0.49	(−0.83, −0.15)	**0.005**	−0.18	(−0.73, 0.38)	0.533	0.87	(0.48, 1.27)	**<0.001**
**Mental health ^b^**									
Poor well-being	−0.22	(−0.97, 0.53)	0.570	0.11	(−0.64, 0.85)	0.774	−0.80	(−1.7, 0.10)	**0.051**
Depression	−0.24	(−1.05, 0.57)	0.559	0.26	(−0.48, 0.99)	0.494	−0.23	(−1.21, 0.75)	0.648
Social anxiety	0.52	(−0.02, 1.07)	**0.059**	0.37	(−0.36, 1.10)	0.324	−0.75	(−1.6, 0.09)	**0.029**

Note: Boldface indicates statistical significance (*p* value < 0.05). BMI, Body mass index; WC, Waist circumference; SBP, Systolic blood pressure; DBP, Diastolic blood pressure; FPG, Fasting plasma glucose; TG, Triglyceride; HDL, High-density lipoprotein; CI, Confidence interval. β × 10: regression coefficient was displayed after multiplying 10. ^a^ Generalized estimating equation using Gamma with log link model with exchangeable structure. ^b^ Generalized estimating equation using Binary logistic model with exchangeable structure. ^c^ Adjusted for sex, age, class, and monthly household income.
